# The response of tumour cells to radiation and cytotoxic drugs--a comparison of clonogenic and isotope uptake assays.

**DOI:** 10.1038/bjc.1984.229

**Published:** 1984-11

**Authors:** P. R. Twentyman, G. A. Walls, K. A. Wright

## Abstract

We have carried out a series of experiments to compare the response to radiation and drugs of cells disaggregated from solid tumours as assayed by clonogenic survival and by an isotope incorporation method. This latter assay consisted of measuring the 24 h uptake of tritium labelled thymidine into cells plated in liquid medium upon a layer of semi-solid agar. The isotope was administered 4 days after plating. For cells from the RIF-1 mouse tumour, good agreement was seen between response to radiation, adriamycin, vincristine and CCNU as measured by the two assays. The two curves for radiation response, for example, showed similar shoulders and subsequent exponential regions. For cells from xenografts of the NCI-H69 human small cell lung cancer line, the response to radiation was dose-related for both assays, but the curve for clonogenic assay was about twice as steep as that for isotope uptake. For a range of five cytotoxic drugs, good agreement was seen between the two assays over the first 1 1/2 decades of response but with a tendency for the isotope uptake curve to plateau with further increasing drug dose. It appears that, at least for these two well-defined experimental tumour systems, the isotope uptake assay can provide a rapid quantitative assessment of cellular drug and radiation sensitivity comparable to that provided by clonogenic assay but in a much shorter period of time.


*Br. J. Cancer* (1984), **50**, 625–631

### The response of tumour cells to radiation and cytotoxic drugs – A comparison of clonogenic and isotope uptake assays

P.R. Twentyman, G.A. Walls & K.A. Wright

*Medical Research Council, Clinical Oncology and Radiotherapeutics Unit, Hills Road, Cambridge CB2 2QH, UK.*

**Summary** We have carried out a series of experiments to compare the response to radiation and drugs of cells disaggregated from solid tumours as assayed by clonogenic survival and by an isotope incorporation method. This latter assay consisted of measuring the 24 h uptake of tritium labelled thymidine into cells plated in liquid medium upon a layer of semi-solid agar. The isotope was administered 4 days after plating. For cells from the RIF-1 mouse tumour, good agreement was seen between response to radiation, adriamycin, vincristine and CCNU as measured by the two assays. The two curves for radiation response, for example, showed similar shoulders and subsequent exponential regions. For cells from xenografts of the NCI-H69 human small cell lung cancer line, the response to radiation was dose-related for both assays, but the curve for clonogenic assay was about twice as steep as that for isotope uptake. For a range of five cytotoxic drugs, good agrement was seen between the two assays over the first $1\frac{1}{2}$ decades of response but with a tendency for the isotope uptake curve to plateau with further increasing drug dose.

It appears that, at least for these two well-defined experimental tumour systems, the isotope uptake assay can provide a rapid quantitative assessment of cellular drug and radiation sensitivity comparable to that provided by clonogenic assay but in a much shorter period of time.

Predictive sensitivity testing of cells from individual patients' tumours has received much recent attention. Much of the impetus has been provided by the development of *in vitro* clonogenic assays for cells taken directly from tumours (Hamburger & Salmon, 1977; Courtenay & Mills, 1978). Although these clonogenic assays have become widely used, and it has been claimed that they can correctly predict clinical response to cytotoxic drugs (Salmon *et al.,* 1978; von Hoff *et al.,* 1981), a number of severe problems remain. Among these are the absolute requirement for a single cell suspension of good viability, the very low plating efficiencies of most tumour specimens (<0.1%), the subjective nature of colony assessment, and the length of time needed to obtain a result (2–4 weeks). Various alternatives to clonogenic assays have been suggested, such as dye exclusion and isotope uptake assays (Dendy *et al.,* 1976; Durkin *et al.,* 1979; Volm *et al.,* 1979; Morgan *et al.,* 1983; Weisenthal *et al.,* 1983; Wilson *et al.,* 1984). Whereas some isotope assays examine very "short term" effects of drug upon specific biochemical processes (Volm *et al.,* 1979; Sanfilippo *et al.,* 1981). others are dependent upon the establishment and proliferation of cells in culture following removal from the tumour (Dendy *et al.,* 1976; Roper & Drewinko, 1976; Morgan *et al.,* 1983; Wilson *et al.,* 1984), in a way which in many respects relates more closely to a clonogenic assay but performed at a much earlier stage of growth. A major problem with such assays has been the potential contribution to proliferation (and hence isotope uptake) of stromal cells present in the tumour and able to proliferate for some time on plastic or glass surfaces. In an attempt to overcome this problem, Friedman & Glaubiger (1982) have described a "liquid top" culture system in which the tumour cells are plated in liquid medium into dishes previously base-coated with a solid layer of agar. The anchorage-dependent stromal cells are unable to proliferate whereas the anchorage-independent tumour cells aggregate and proliferate. Using this system, Friedman & Glaubiger (1982) showed that, for a variety of human tumour samples, good agreement was seen for predictions of drug sensitivity or resistance between the isotope uptake assay and the human tumour stem cell assay of Hamburger & Salmon (1977).

In order to obtain more precise quantitative data regarding the relationship between this [$^3$H]TdR uptake assay and clonogenic assay, we have carried out experiments to obtain radiation and drug dose-response curves for cells taken from an established mouse tumour (RIF-1) and xenografts of the human small cell lung cancer line NCI-H69. For both tumour types, responses have been measured using both assay systems and the results compared.

Correspondence: P.R. Twentyman
Received 19 March 1984; accepted 12 July 1984.

© The Macmillan Press Ltd., 1984

### Materials and methods

### *Tumours*

The RIF-1 mouse sarcoma grows both as a solid tumour in the C3H mouse and as a monolayer in culture (Twentyman *et al.*, 1980). Tumours were initiated by the inoculation of $2 \times 10^5$ cells from culture into the gastrocnemius muscle of the hind leg and reached a volume of $500\,mm^3$ at 11–12 days after injection. The host cell component of RIF-1 tumours has been measured at around 50% (Dr D.W. Siemann, personal communication). The human small cell lung cancer line NCI-H69 (kindly supplied by Dr Desmond Carney) was maintained in culture by weekly passage and xenografts were initiated by the inoculation of $2–5 \times 10^6$ cells either from culture or from a disaggregated xenograft tumour into the gastrocnemius muscle of MF1 nude mice. Tumours reached a volume of $500\,mm^3$ at 2–4 weeks after injection. Histological examination of tumour sections showed tightly packed round cells with little cytoplasm and relatively little stroma.

For each response experiment, a pair of similar tumours was excised under sterile conditions and finely minced with scissors. The fragments were then agitated for 1 h in culture medium containing $1\,mg\,ml^{-1}$ of neutral protease (Sigma Type IX) (Twentyman & Yuhas, 1980). At the end of this time, the material was filtered through cotton gauze, and centrifuged at 200 *g* for 5 min. The pellet was resuspended in medium and a haemocytometer count performed on the resulting single cell suspension. Appropriate dilutions were then prepared for treatment with radiation or drugs.

### *Treatments*

For radiation treatments, a cell suspension was prepared in 6 ml Hanks balanced salt solution at $10^7\,cells\,ml^{-1}$. The suspension was placed in a $25\,cm^2$ tissue culture flask on the surface of a bucket of crushed ice. The flask was then exposed to incremental doses of 250 kV X-rays and, after each increment, 0.5 ml of the suspension was removed, placed into a plastic tube, and stored on crushed ice. At the end of the radiation treatment cells from the various groups were assayed for response as below.

For drug treatments, freshly prepared drugs in volumes of 10–200 $\mu l$ were added to plastic tubes containing $10^6$ cells in 5 ml of the appropriate culture medium. The media used for RIF-1 and NCI-H69 cells were Eagles MEM with 20% new born calf serum and modified Hams F12 with 15% foetal calf serum respectively (see below). Adriamycin (Farmitalia Ltd.) was dissolved in water, melphalan (Chester Beatty Research Institute) was dissolved in acidified ethanol, vincristine (Eli Lilly) was dissolved in water, CCNU (U.S. National Cancer Institute) was dissolved in absolute ethanol and nitrogen mustard (mustine hydrochloride, Boots) was dissolved in water. The tubes were then incubated for 1 h at 37°C with intermittent agitation. At the end of this period, the cells were rinsed twice by centrifugation (5 min at 200 *g*) and resuspension and finally resuspended in medium. Appropriate dilutions were then prepared for the response assays as below.

### *Clonogenic assays*

Cells from RIF-1 tumours were assayed for clonogenic survival as previously described (Twentyman *et al.*, 1980) but with some modifications. Varying numbers of cells were plated into 90 mm plastic petri dishes (Sterlin Ltd.) containing a total of 11 ml of Eagles MEM with 20% new born calf serum and supplemented with penicillin and streptomycin. Dishes were incubated at 37°C for 13 days in an atmosphere of 8% $CO_2$ and 92% air. At the end of this time, the dishes were rinsed in saline and stained in a solution of crystal violet in methanol. Colonies containing at least 50 cells were counted under a binocular dissecting microscope. The routine plating efficiency of RIF-1 cells was in the range 12–27%.

The clonogenic assay used for NCI-H69 cells from xenograft tumours in nude mice was essentially that of Courtenay & Mills (1978) in which low oxygen tension and the addition of rat red cells were used to optimise clonogenicity.

The medium used in this assay was modified Hams F12 supplemented with 15% foetal calf serum and with penicillin and streptomycin (all supplied by Gibco Biocult Ltd.) Red blood cells from August rats were obtained by cardiac puncture using preservative-free heparin, separated by centrifugation, rinsed 3 times with phosphate-buffered saline (PBS) and resuspended to the original blood volume in medium. The red cell suspension was then heated to 44°C for 1 h and stored at 4°C for up to one month. A 1/8 dilution in medium was carried out immediately before use of the red cells. A 6% solution of Agar Noble (Difco) in water was prepared and sterilized by boiling for 15 min. This was then diluted 1/10 in prewarmed medium (44°C) to give a final concentration of 0.6% and the solution kept at 44°C until required. Suspensions of the test cells in medium were prepared at 2.5 × the required final concentration and kept at 37°C.

For cloning, 2.0 ml of the cell suspension was added to 0.5 ml of red cell suspension followed by 2.5 ml of 0.6% agar solution. Aliquots of 1 ml of this suspension were then placed into each of 3 or 4 sterile plastic tubes (Falcon Plastics, No. 2051). These tubes were stood in crushed ice until the agar set. They were then each gassed for 6 seconds with a mixture of 90% nitrogen, 5% oxygen and 5% carbon dioxide and the top of each tube "snapped" closed. The tubes were placed in racks in plastic cake boxes which were then gassed with the same mixture for 10 min before being sealed and incubated at 37°C. After 7 and 14 days of incubation, 1 ml of medium was added to the agar plug in each tube and the tubes and boxes regassed.

At the end of the 21-day incubation period, the agar plug was tipped out from each tube into the inverted lid of a 5 cm plastic petri dish. The base of the dish was then pushed down onto the plug so that the agar spread in a thin layer. Colonies containing more than 50 cells were counted under an inverted microscope. The plating efficiency of cells from NCI-H69 xenografts was usually between 30 and 60% but values as low as 18% and as high as 92% have occurred in single experiments.

### *Isotpe uptake assay*

The tritium-labelled thymidine ([$^3$H]TdR) uptake assay used was essentially that of Friedman & Glaubiger (1982) but with different media. Experiments were carried out using 24-well plates (Falcon) each well being 16 mm in diameter. Bottom layers containing Difco Noble Agar at a concentration of 0.5% were prepared by boiling a solution of 5% agar in water for 10 min and then diluting 1/10 with medium and holding at 44°C. Aliquots of 0.5 ml were then pipetted into each well and these were stored in the refrigerator in plastic boxes gassed with 5% $CO_2$/95% air until required. Eagles medium with 20% new born calf serum was used in both the bottom (agar) layer and the top (liquid) layer for experiments with RIF-1 cells. For NCI-H69 cells, the bottom layer contained RPMI 1640 medium with 10% foetal calf serum while the top layer contained modified Ham's F12 medium with 15% foetal calf serum. RPMI 1640 is the standard medium used in this laboratory for the growth of continuous cell lines of human small cell lung cancer. Liquid top layers consisted of 1 ml of medium containing the appropriate number of control or treated cells. Preliminary experiments were carried out in which different numbers of control cells were plated out so as to establish the range of linearity of the system (see Results section). In response experiments, $10^4$ RIF-1 cells were used and, for NCI-H69, assays were carried out using both $5 \times 10^4$ and $10^4$ cells. Wells with 1 ml of liquid medium but no cells were also included as controls.

Well plates containing RIF-1 cells were then placed in a humidified gassing incubator (8%$CO_2$ 92% Air) at 37°C; while those containing NCI-H69 cells were incubated at 37°C in plastic boxes gassed with 5% $O_2$, 5% $CO_2$ and 90% nitrogen. After 4 days, 0.5–2 $\mu$Ci [$^3$H]TdR (49 Ci mmol$^{-1}$, Amersham International) was added to each well in 20 $\mu$l PBS. The well was then incubated for a further 24 h. At the end of that time, the liquid medium containing the cells was removed from the surface of each well with a Pasteur pipette and transferred to a plastic centrifuge tube containing 5 ml of PBS. The agar was then rinsed twice with 1 ml of PBS and the washes added to the tube. The sample was centrifuged for 10 min at 200 *g*, the supernatant decanted and the pellet resuspended in 3 ml of 5% trichloroacetic acid (TCA) in order to precipitate protein and nucleic acids. After standing on ice for 30 min the sample was recentrifuged for 10 min at 500 *g*. All subsequent centrifugations were performed at 500 *g* for 10 min. The supernatant was decanted and the pellet resuspended in 3 ml of TCA. After 10 min on ice the sample was recentrifuged, the supernatant decanted and the pellet resuspended in 3 ml of absolute methanol. This suspension was recentrifuged, the supernatant decanted and 0.5 ml of hyamine hydroxide (methylbenzethonium hydroxide, 1 M in methanol, Sigma) was added. The sample was heated in a water bath at 60°C to dissolve the pellet and then transferred using a Pasteur pipette to a 5 ml plastic liquid scintillation counting insert vial containing 4 ml of Aquasol-2 (New England Nuclear). The sample tube was washed twice with about 0.5 ml of Aquasol-2 from the insert vial and these washes returned to the vial. The insert vial was placed into a glass counting vial and stored in the dark 24 h before counting on a Nuclear Chicago Isocap 300 liquid scintillation counter.

### Results

### *Linearity*

Figure 1 shows typical data demonstrating a linear relationship between the number of cells plated and the amount of [$^3$H]TdR incorporated into TCA-precipitable material. Linearity was seen with RIF-1 cells over the range of $10^3$–$10^5$ cells and with NCI-H69 over the range of $10^3$–$5 \times 10^4$ cells. For subsequent response experiments, we used $10^4$ cells for RIF-1 and both $5 \times 10^4$ and $10^4$ cells for NCI-H69.

### *RIF-1 experiments*

Data for radiation and cytotoxic drug response of cells from RIF-1 mouse tumours are shown in Figures 2 and 3 respectively. The radiation response curves (Figure 2) for the two assays were essentially identical, showing initial shoulders and subsequent exponential falls, although at each dose the isotope uptake point is above the corresponding survival

**Figure 1** Relationship between [$^3$H]TdR uptake and number of cells plated per well in the 1 ml liquid top layer: (●) RIF-1 cells ([$^3$H]TdR = 2.0 $\mu$ Ci/well) (▲) NCI-H69 cells ([$^3$H]TdR = 0.5 $\mu$Ci/well).

**Figure 2** Change in cell surviving fraction (●) or in [$^3$H]TdR uptake as fraction of control (○) of RIF-1 cells treated with different doses of X-rays. The line is fitted by eye to the surviving fraction data only.

**Figure 3** Change in cell surviving fraction (closed symbols) or in [$^3$H]TdR uptake as fraction of control (open symbols) of RIF-1 cells treated for 1 h with different doses of cytotoxic drugs. CCNU (●, ○); adriamycin (▲, △); vincristine (■, □). The lines are fitted by eye to the surviving fraction data only.

point. The line fitted by eye to the surviving fraction data has a slope ($D_0$) of 1.76 Gy and an extrapolation number ($n$) of 4. For the cytotoxic drugs (Figure 3), there was essentially no response to vincristine (VCR) at any one of the doses used for either assay. There was little or no effect of adriamycin (ADM) at the three lowest doses but a considerable response for both assays at 5 $\mu$g ml$^{-1}$. A progressive response with increasing dose of CCNU was seen in both assays. The excellent agreement between the two assay systems seen in these preliminary experiments provided impetus to investigate the human lung cancer xenograft response.

*NCI-H69 xenograft experiments*

The response of cells from NCI-H69 xenografts to radiation and cytotoxic drugs in the two assay systems are shown in Figure 4 (a–c). The results shown are for $5 \times 10^4$ cells per well. The results for $10^4$ cells per well were essentially identical. The experiments for radiation, ADM and melphalan (MEL) were carried out twice and gave closely similar results to those shown. The radiation

**Figure 4** Change in cell surviving fraction (closed symbols) or in [$^3$H]TdR uptake as fraction of control (open symbols) of NCI-H69 cells treated with different doses of X-rays (4a) or cytotoxic drugs (4b and 4c). In 4b, adriamycin (●, ○); melphalan (▲, △). In 4c, vincristine (●, ○); nitrogen mustard (▲, △); CCNU (■, □). The lines are fitted by eye to the surviving fraction data only.

response curves are somewhat different for the two assay systems (Figure 4a). For the clonogenic assay there is a small initial shoulder with a subsequent exponential fall characterised by a $D_0$ of $\sim$0.72 Gy. The repeat experiment yielded a curve with an almost identical slope but without an initial shoulder. For the isotope uptake assay the response curve has little, if any, shoulder and is less steep. A line fitted by eye to the points in the region between 1 Gy and 6 Gy has a slope corresponding to a $D_0$ of 1.50 Gy. The slope of the response curve decreases at higher radiation doses at a level of isotope uptake of $\sim$1% of control. The results for ADM and MEL (Figure 4b) show excellent agreement between the two assays for the first $1\frac{1}{2}$ decades of response but with a tendency for the isotope uptake response to plateau at around 2 decades and therefore no longer be dose-related. This same tendency was also seen for nitrogen mustard (HN2) and CCNU (Figure 4c). The responses to vincristine (VCR) (Figure 4c) were in particularly good agreement over the two decades of response measured.

The data from Figure 4 together with those from repeat experiments (not shown) have been combined in Figure 5 to demonstrate the relationship between the surviving fraction and the [$^3$H]TdR uptake for all agents studied. The points for radiation response are fitted by a curve which indicates a rather greater response in terms of cell survival than in terms of isotope uptake at all dose levels. The curve fitted to all the drug data is initially more shallow than the radiation curve, indicating a somewhat greater effect on isotope uptake, but becoming very steep at higher drug doses when isotope uptake tends to plateau at around 2 decades of response. We have not shown a plot similar to Figure 5 for the RIF-1 data because of the very close correspondence of the 2 assays for both radiation and drugs in this tumour. Under these circumstances a plot similar to Figure 5 would show all points lying on or close to the line of equivalence (i.e. slope = 1.0).

### Discussion

There are remarkably few papers in the literature where the results of clonogenic and non-clonogenic assays have been directly compared in well-characterised experimental systems. In general, isotope uptake assays can be divided into "short term assays" which measure the effect of cytotoxic drugs on specific biochemical processes (e.g. DNA or protein synthesis) within a very few hours of removal from the tumour (Volm *et al.*, 1979; Sanfilippo *et al.*, 1981) and those assays which

**Figure 5** Relationship between cell surviving fraction and [$^3$H]TdR uptake as fraction of control of NCI-H69 cells treated with various cytotoxic agents. Symbols are defined in the key. The solid line is fitted by eye to the radiation response data and the broken line to the data for a range of cytotoxic drugs.

depend upon the rate of proliferation of cells established in culture some days after removal and treatment. In this discussion, we will only consider the latter type of assay as it is clearly more akin to a clonogenic assay but performed at a much earlier stage of growth. A study by Roper & Drewinko (1976) examined the effect of cytotoxic drugs on the clonogenicity and the [$^3$H]TdR incorporation of cells from a mouse lymphoma cell line. They carried out the isotope uptake measurements at various intervals from 1–5 days. Their conclusion was that isotope uptake values were not clearly dose-dependent at any given time-interval and did not correlate with colony formation. More recently Rupniak *et al.* (1983) compared cell survival in a clonogenic assay and [$^3$H]TdR labelling index changes after drug treatment of a murine cell line. Their 24 h isotope labelling periods were either 24–48 h or 48–72 h after plating. For the four drugs examined, the labelling index was generally reduced in circumstances where the clonogenic fraction was reduced although it was not possible to define a quantitative relationship between the two assays.

Two recent studies have made a less direct comparison of drug sensitivity in cells both treated and assayed under different conditions. Morgan *et al.* (1983) compared the sensitivity of short-term cultures of human gliomas to various drugs by determining the drug doses necessary to depress either clonogenicity or monolayer [$^3$H] leucine incorporation by 50 or 90%. A high degree of correlation between the 2 methods was seen when comparing $ID_{50}$ values but not when comparing $ID_{90}$ values. In a comparison of a clonogenic assay and a monolayer isotope uptake assay, Wilson *et al.* (1984) showed for 2 cell lines that although the assays did not produce identical dose-response data they each showed a dose-dependent response. For each assay it was possible, on the basis of retrospective clinical response data, to choose a cut-off point for response which made the assay valid for further predictive testing.

In the original report by Friedman & Glaubiger (1982) which first described the "liquid top" assay which we have used, they compared isotope uptake results with clonogenic assay for 61 drug tests on human cell lines and clinical tumour samples. They state that drug resistance/sensitivity determinations (defined by arbitrary but consistent criteria) agreed in 54 of 61 determinations, and furthermore that in 22 experiments in which drug sensitivity curves (2 or 3 dose levels) were compared 21 were "similar" in both assay systems. Very recently Friedman *et al.* (1983) have used their system to obtain dose-effect curves for the radiation response of a Chinese hamster ovary cell line and found the results to be closely similar to those obtained with clonogenic assay. Furthermore dose-effect relationships were obtained for the radiation response of 3 primary human tumour biopsy specimens and these showed a conventional exponential fall. These data together with those which we report in this paper appear to indicate that, for $1\frac{1}{2}$–2 decades of response, the [$^3$H]TdR "liquid top" assay produces better agreement with clonogenic assay (for the same treated population of tumour cells) than has been previously reported. Although agreement over 3 or more decades of response would be preferable, it must be remembered that most currently used clonogenic assays for predictive testing rely upon a reduction of colonies to only 50% or 30% of the initial value in order to predict sensitivity (Salmon *et al.*, 1978; Von Hoff *et al.*, 1981). The extremely low plating efficiency of most human tumour biopsy specimens means that study over more than 1 decade of response is generally impractical. There are clearly differences between the assays in that, for instance, the radiation and drug data in Figure 5 are fitted by somewhat different lines. It is certainly not, of course, to be expected that any two response assays will provide identical dose-effect curves over a wide range of responses and agents. Whereas the clonogenic assay will measure only the long-term survival of cells in a "yes or no" manner, the isotope uptake assay will additionally reflect the various lengths of division delay induced by different agents and also any changes in the subsequent proliferation rate of surviving cells. If, for instance, the mean colony size is reduced by a given treatment, this factor will not be accounted for in a clonogenic assay but would be expected to produce a reduced isotope uptake. To what extent the various aspects of "response" are involved in determining the relatively short term clinical responses used to validate predictive testing remains a matter of speculation. Even in well-established experimental animal tumours the relationship between tumour growth delay and clonogenic cell survival is complicated by a very wide range of factors (Twentyman, 1980). What is required in assessing the comparative validity of two response assays is that each assay be able to provide a quantitative dose-effect relationship over a similar range of doses. We believe that the [$^3$H]TdR "liquid top" assay fulfils this requirement when compared with clonogenic cell survival.

Our further studies are aimed at optimising the media, and growth conditions for a variety of primary human types and also determining the contribution of non-tumour cells present in clinical specimens to isotope uptake under the conditions of the assay. Should the initial promise of the method stand up to further investigation, the "liquid top" assay, producing results within 6 days, should prove a useful tool in the determination of human tumour sensitivity characteristics.

We thank Dr Paul Workman for providing the xenografted tumours used in this study, and Professor N.M. Bleehen for his continuing interest and discussion of this work.

**References**

COURTENAY, V.D. & MILLS, J. (1978). An *in vitro* colony assay for human tumours grown in immune-suppressed mice and treated *in vivo* with cytotoxic agents. *Br. J. Cancer*, **37**, 261.

DENDY, P.P., DAWSON, M.P.A., WARNER, D.M.A. & HONESS, D.J. (1976). Quantitative assays of *in vitro* drug damage. In: *Short Term Culture of Human Tumours*. (Ed. Dendy), New York: Academic Press, p. 139.

DURKIN, W.J., GHANTA, V.K., BALCH, C.M., DAVIS, D.W. & HIRAMOTO, R.N. (1979). A methodological approach to the prediction of anti-cancer drug effect in humans. *Cancer Res.*, **39**, 402.

FRIEDMAN, H.M. & GLAUBIGER, D.L. (1982). Assessment of *in vitro* drug sensitivity of human tumour cells using [$^3$H] thymidine incorporation in a modified human tumour stem cell assay. *Cancer Res.*, **42**, 4683.

FRIEDMAN, H.M., CANDILIS, P., JOHNSON, G. & GLAUBIGER, D.L. (1983). *In vitro* radiation survival curves from human tumour cell lines and primary human tumour surgical explants using a [$^3$H] thymidine assay. *Proc. AACR 1983*, p. 316.

HAMBURGER, A. & SALMON, S.E. (1977). Primary bioassay of human tumor stem cells. *Science*, **197**, 461.

MORGAN, D., FRESHNEY, R.I., DARLING, J.L., THOMAS, D.G.T. & CELIK, F. (1983). Assay of anticancer drugs in tissue culture: cell cultures of biopsies from human astrocytoma. *Br. J. Cancer*, **47**, 205.

ROPER, P.R. & DREWINKO, B. (1976). Comparison of *in vitro* methods to determine drug-induced cell lethality. *Cancer Res.*, **36**, 2182.

RUPNIAK, H.T., DENNIS, L.Y. & HILL, B.T. (1983). An intercomparison of *in vitro* assays for assessing cytotoxicity after a 24 hour exposure to anti-cancer drugs. *Tumori*, **69**, 37.

SALMON, S.E., HAMBURGER, A.W., SOEHNLEN, B., DURIE, B.G.M., ALBERTS, D.S. & MOON, T.E. (1978). Quantitation of differential sensitivity of human-tumour stem cells to anti-cancer drugs. *N. Engl. J. Med.*, **298**, 1321.

SANFILIPPO, O., DAIDONE, M.G., COSTA, A., CANETTA, R. & SILVESTRINI, R. (1981). Estimation of differential *in vitro* sensitivity of non-Hodgkin lymphomas to anticancer drugs. *Eur. J. Cancer*, **17**, 217.

TWENTYMAN, P.R. (1980). Experimental chemotherapy studies: intercomparison of assays. *Br. J. Cancer*, **41**, (Suppl. IV), 279.

TWENTYMAN, P.R. & YUHAS, J.M. (1980). Use of a bacterial neutral protease for disaggregation of mouse tumours and multicellular tumour spheroids. *Cancer Lett.*, **9**, 225.

TWENTYMAN, P.R., BROWN, J.M., GRAY, J.W., FRANKO, A.J., SCHOLES, M.A. & KALLMAN, R.F. (1980). A new mouse tumour model system (RIF-1) for comparison of end-point studies. *J. Natl Cancer Inst.*, **64**, 595.

VOLM, M., WAYSS, K., KAUFMANN, M. & MATTERN, J. (1979). Pretherapeutic detection of tumour resistance and the results of tumour chemotherapy. *Eur. J. Cancer*, **15**, 983.

VON HOFF, D.D., CASPER, J., BRADLEY, E., SANDBACH, J., JONES, D. & MAKUCH, R. (1981). Association between human tumour colony-forming assay results and response of an individual patients' tumour to chemotherapy. *Am. J. Med.*, **70**, 1027.

WEISENTHAL, L.M., MARSDEN, J.A., DILL, P.L. & MACALUSO, C.K. (1983). A novel dye exclusion method for testing *in vitro* chemosensitivities of human tumours. *Cancer Res.*, **43**, 749.

WILSON, A.P., FORD, C.H.J., NEWMAN, C.E. & HOWELL, A. (1984). A comparison of three assays used for the *in vitro* chemosensitivity testing of human tumour. *Br. J. Cancer*, **49**, 57.


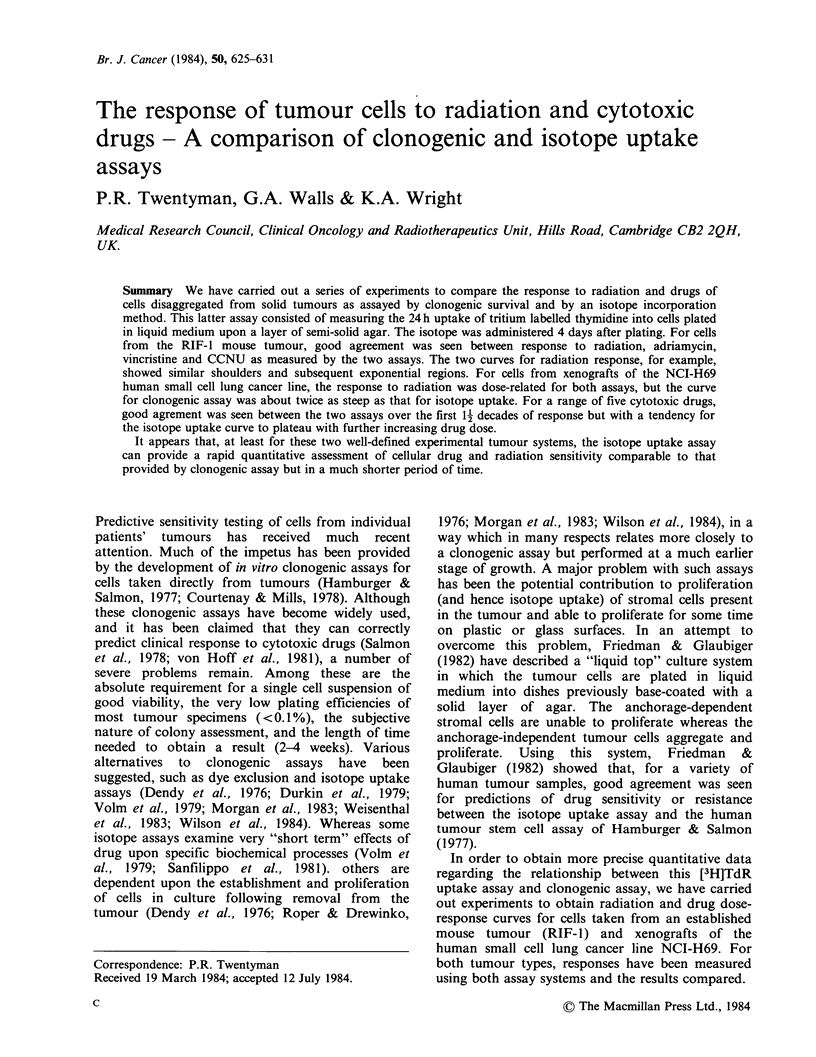

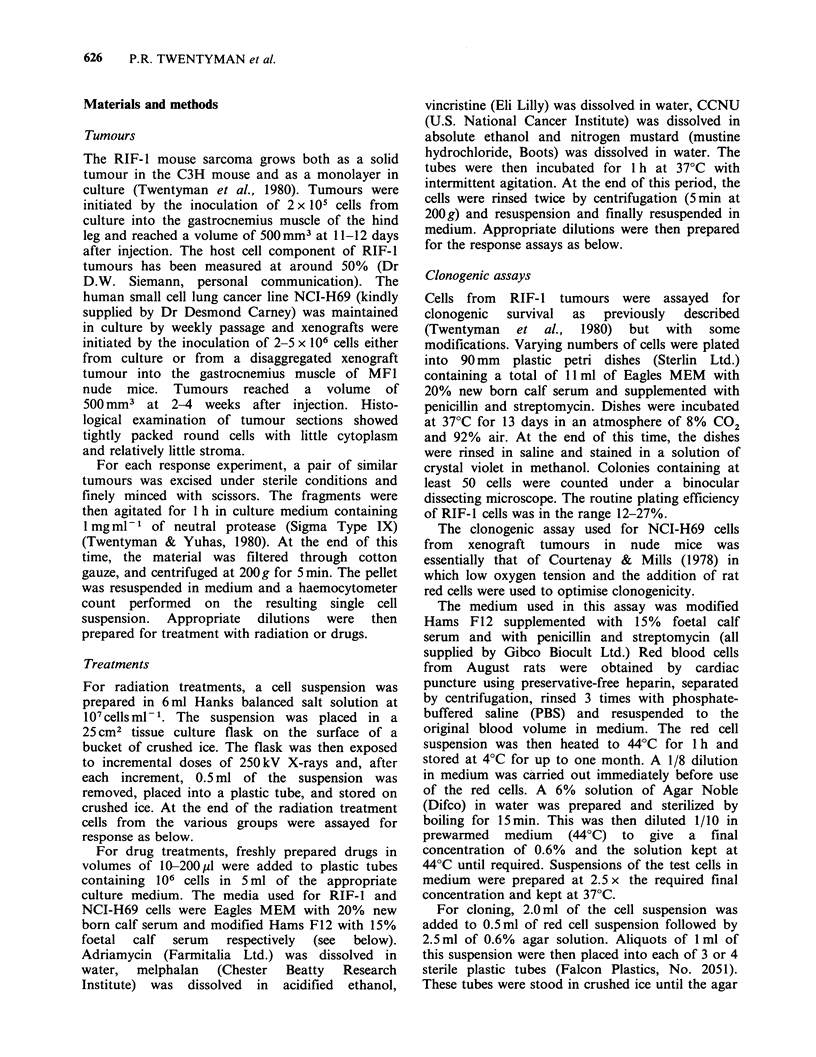

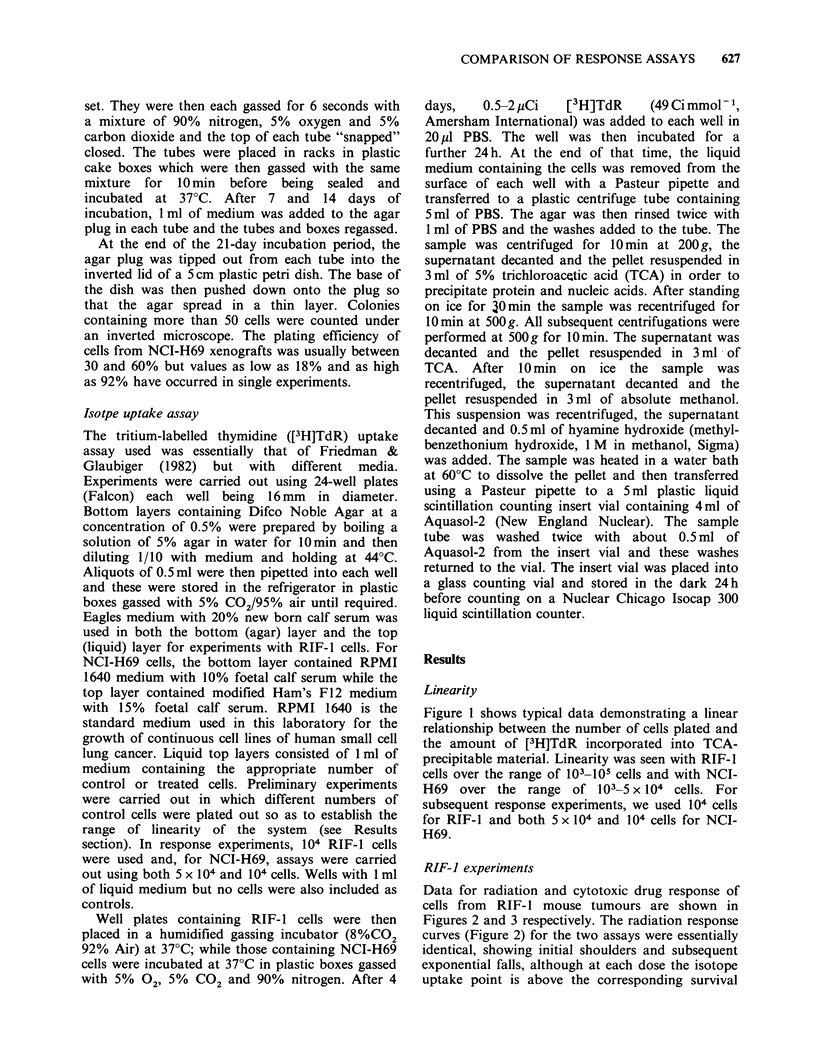

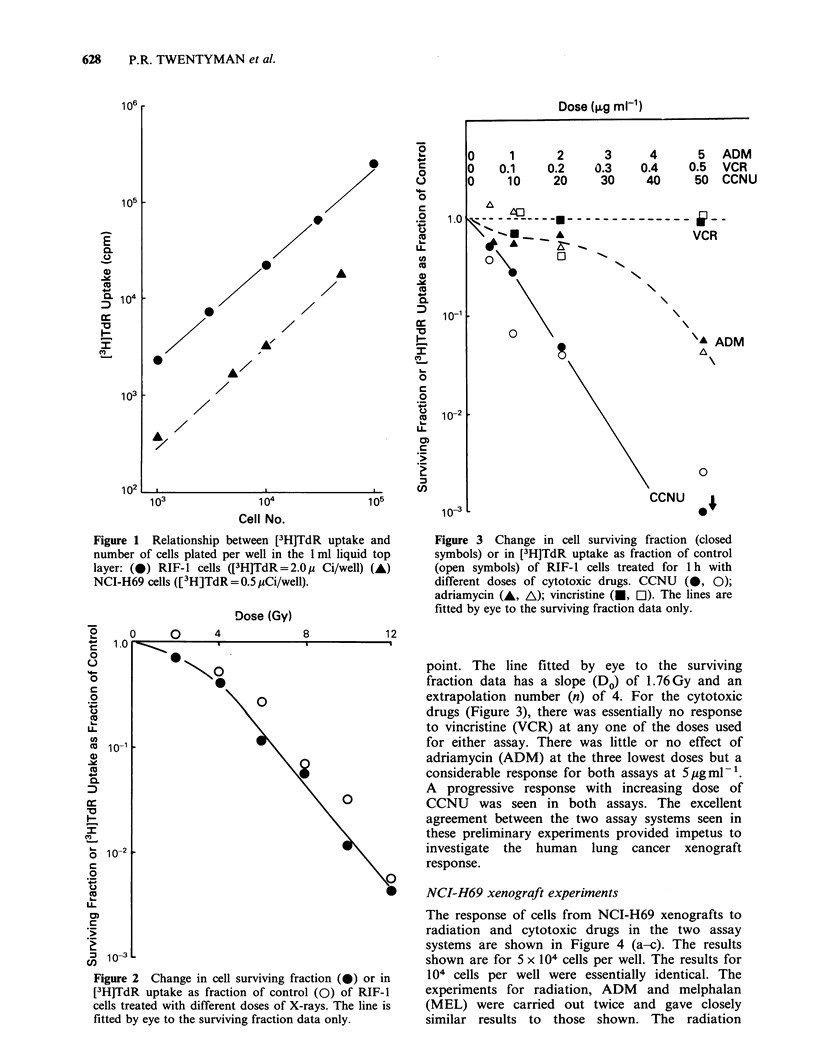

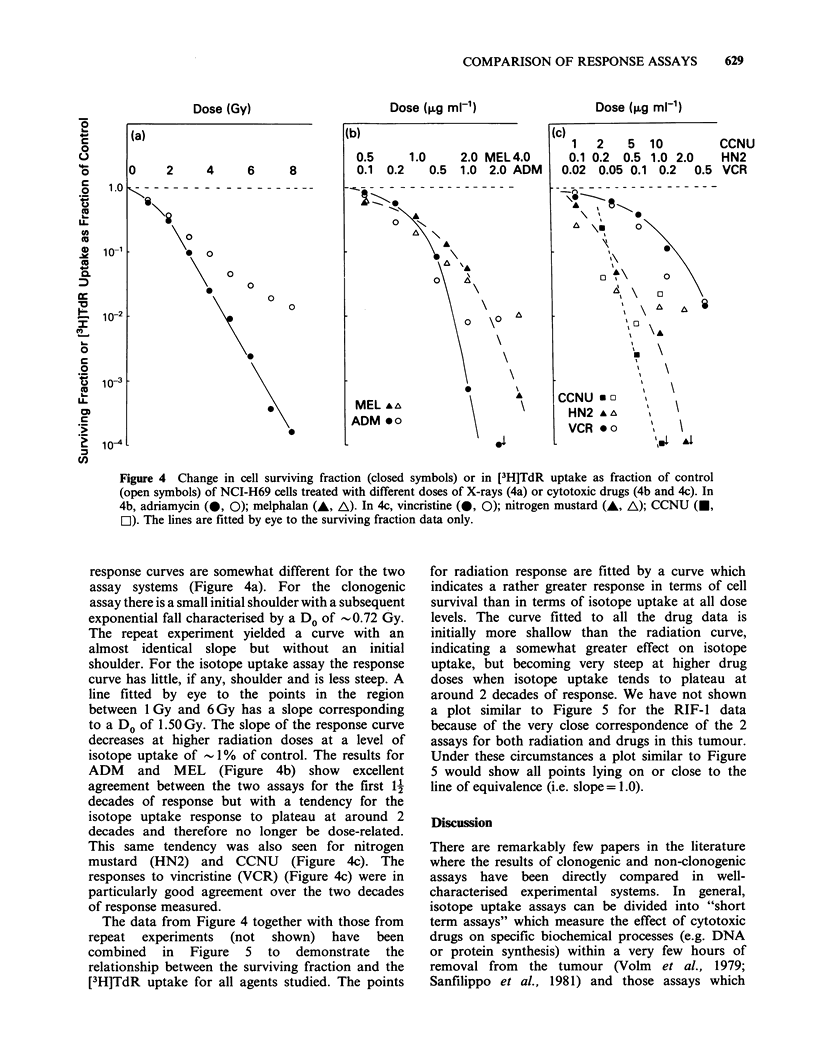

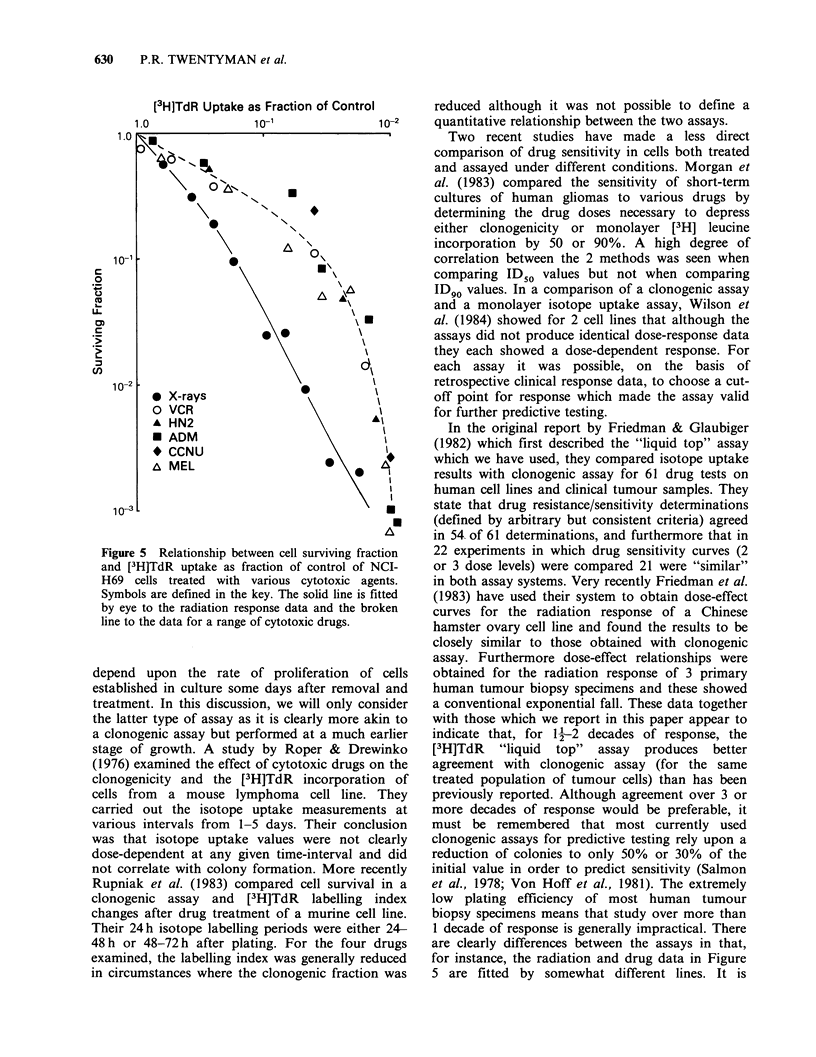

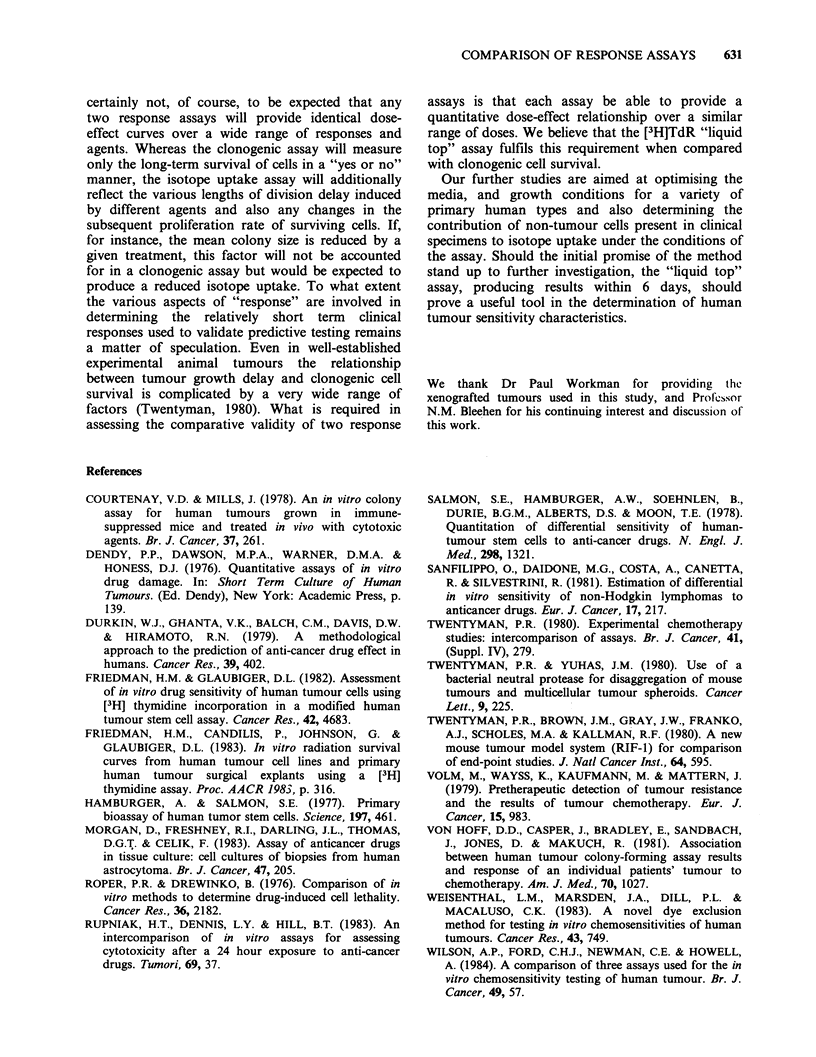


## References

[OCR_01025] Courtenay V. D., Mills J. (1978). An in vitro colony assay for human tumours grown in immune-suppressed mice and treated in vivo with cytotoxic agents.. Br J Cancer.

[OCR_01038] Durkin W. J., Ghanta V. K., Balch C. M., Davis D. W., Hiramoto R. N. (1979). A methodological approach to the prediction of anticancer drug effect in humans.. Cancer Res.

[OCR_01044] Friedman H. M., Glaubiger D. L. (1982). Assessment of in vitro drug sensitivity of human tumor cells using [3H]thymidine incorporation in a modified human tumor stem cell assay.. Cancer Res.

[OCR_01057] Hamburger A. W., Salmon S. E. (1977). Primary bioassay of human tumor stem cells.. Science.

[OCR_01061] Morgan D., Freshney R. I., Darling J. L., Thomas D. G., Celik F. (1983). Assay of anticancer drugs in tissue culture: cell cultures of biopsies from human astrocytoma.. Br J Cancer.

[OCR_01067] Roper P. R., Drewinko B. (1976). Comparison of in vitro methods to determine drug-induced cell lethality.. Cancer Res.

[OCR_01072] Rupniak H. T., Dennis L. Y., Hill B. T. (1983). An intercomparison of in vitro assays for assessing cytotoxicity after a 24 hour exposure to anti-cancer drugs.. Tumori.

[OCR_01078] Salmon S. E., Hamburger A. W., Soehnlen B., Durie B. G., Alberts D. S., Moon T. E. (1978). Quantitation of differential sensitivity of human-tumor stem cells to anticancer drugs.. N Engl J Med.

[OCR_01085] Sanfilippo O., Daidone M. G., Costa A., Canetta R., Silvestrini R. (1981). Estimation of differential in vitro sensitivity of non-Hodgkin lymphomas to anticancer drugs.. Eur J Cancer.

[OCR_01102] Twentyman P. R., Brown J. M., Gray J. W., Franko A. J., Scoles M. A., Kallman R. F. (1980). A new mouse tumor model system (RIF-1) for comparison of end-point studies.. J Natl Cancer Inst.

[OCR_01091] Twentyman P. R. (1980). Experimental chemotherapy studies: intercomparison of assays.. Br J Cancer Suppl.

[OCR_01096] Twentyman P. R., Yuhas J. M. (1980). Use of bacterial neutral protease for disaggregation of mouse tumours and multicellular tumor spheroids.. Cancer Lett.

[OCR_01108] Volm M., Wayss K., Kaufmann M., Mattern J. (1979). Pretherapeutic detection of tumour resistance and the results of tumour chemotherapy.. Eur J Cancer.

[OCR_01114] Von Hoff D. D., Casper J., Bradley E., Sandbach J., Jones D., Makuch R. (1981). Association between human tumor colony-forming assay results and response of an individual patient's tumor to chemotherapy.. Am J Med.

[OCR_01121] Weisenthal L. M., Marsden J. A., Dill P. L., Macaluso C. K. (1983). A novel dye exclusion method for testing in vitro chemosensitivity of human tumors.. Cancer Res.

[OCR_01127] Wilson A. P., Ford C. H., Newman C. E., Howell A. (1984). A comparison of three assays used for the in vitro chemosensitivity testing of human tumours.. Br J Cancer.

